# Supplementary material for: Exploring the Potential of Sulfur Moieties in Compounds Inhibiting Steroidogenesis
Source: Biomolecules. 2023 Sep 5;13(9):1349. doi: 10.3390/biom13091349 (PMC10526780; doi:10.3390/biom13091349)
Supplement: Supplementary file 1 [file biomolecules-13-01349-s001.zip › biomolecules-2562164-supplementary.pdf]

Exploring the potential of sulfur moieties in compounds inhibiting steroidogenesis

Tomasz M. Wróbel, Katyayani Sharma, Iole Mannella, Simonetta Oliaro-Bosso, Patrycja Nieckarz, Therina du Toit, Clarissa Daniela Voegel, Maria Natalia Rojas Velazquez, Jibira Yakubu, Anna Matveeva, Søren Therkelsen, Flemming Steen Jørgensen, Amit V Pandey, Agnese C. Pippione, Marco L. Lolli, Donatella Boschi and Fredrik Björkling

SUPPLEMENTARY INFORMATION

Contents

NMR data.......2
MS data.......10
HPLC data.......14

NMR data

3a

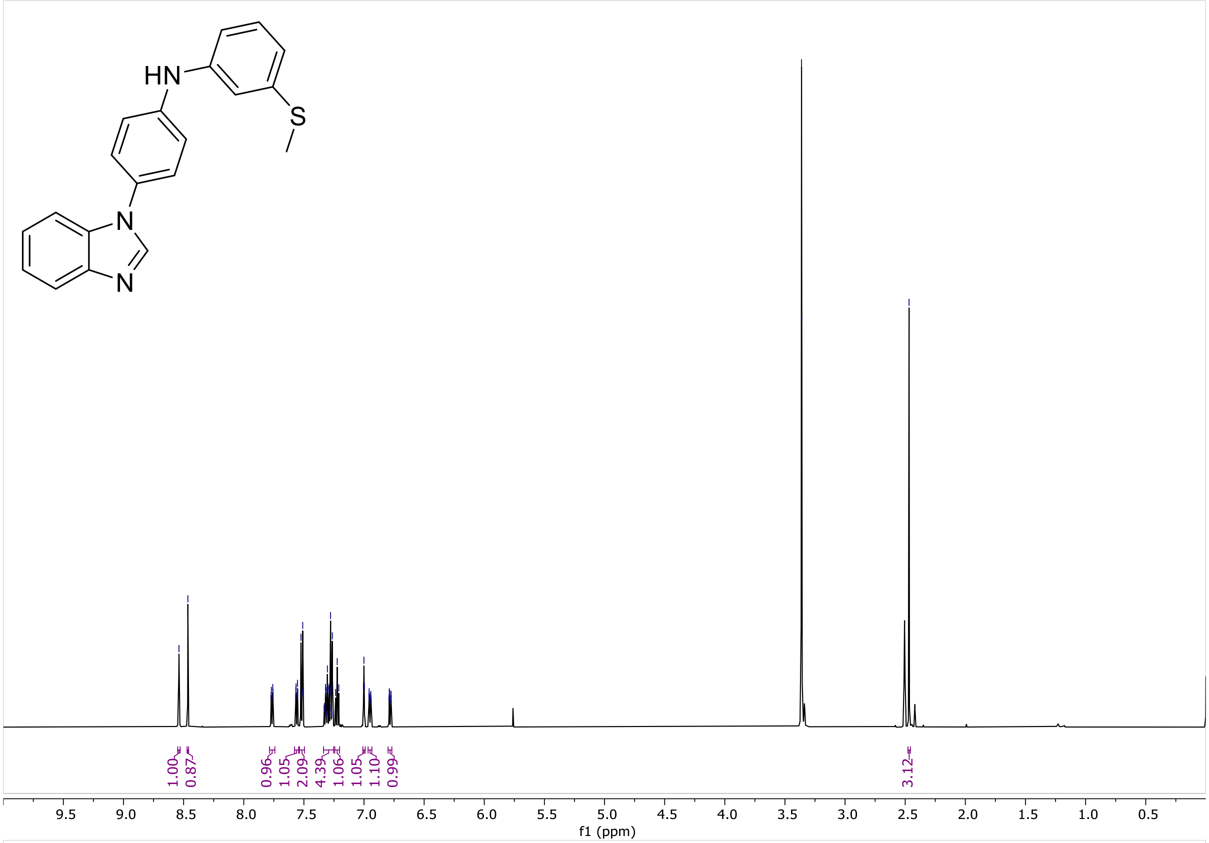
HN
S
N
N
1.00
0.87
0.96
1.05
2.09
4.39
1.06
1.05
1.10
0.99
3.12
9.5 9.0 8.5 8.0 7.5 7.0 6.5 6.0 5.5 5.0 4.5 4.0 3.5 3.0 2.5 2.0 1.5 1.0 0.5
f1 (ppm)

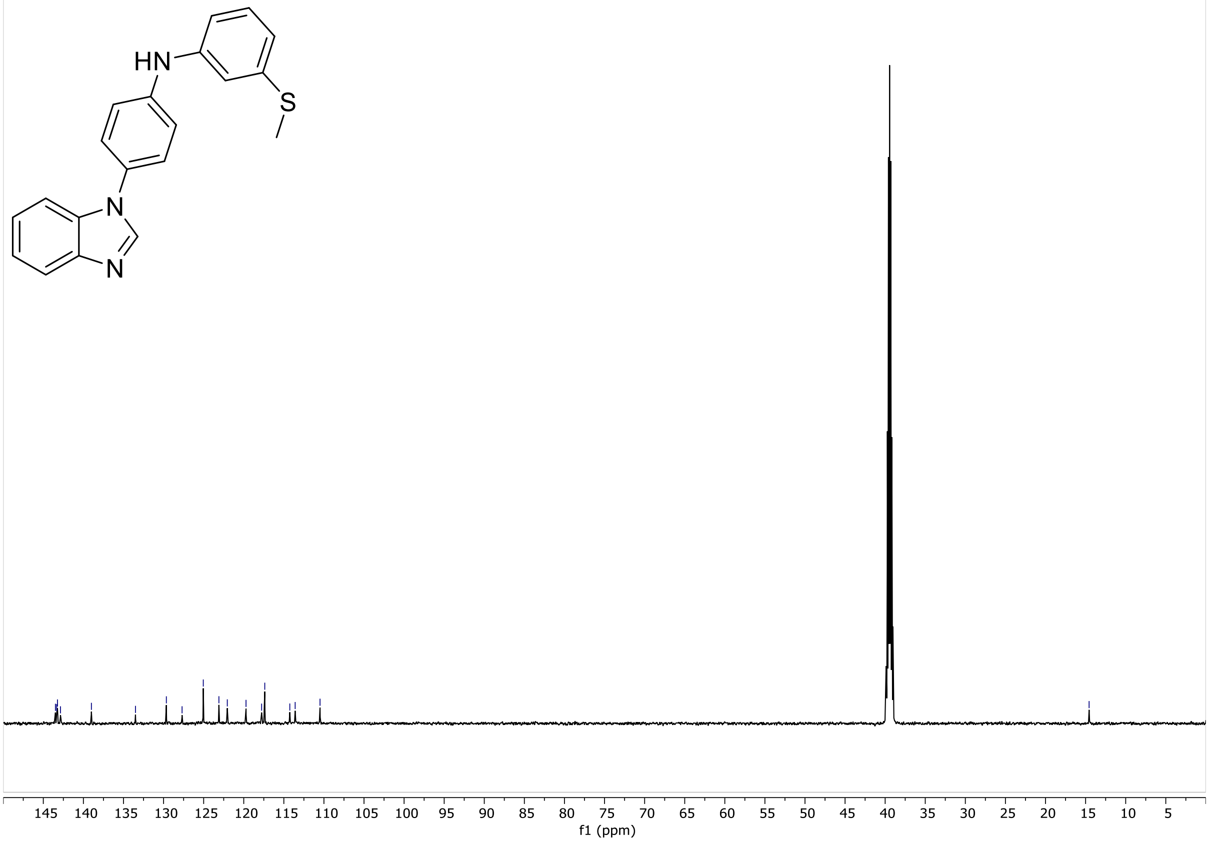
HN
S
N
N
145 140 135 130 125 120 115 110 105 100 95 90 85 80 75 70 65 60 55 50 45 40 35 30 25 20 15 10 5
f1 (ppm)

3b

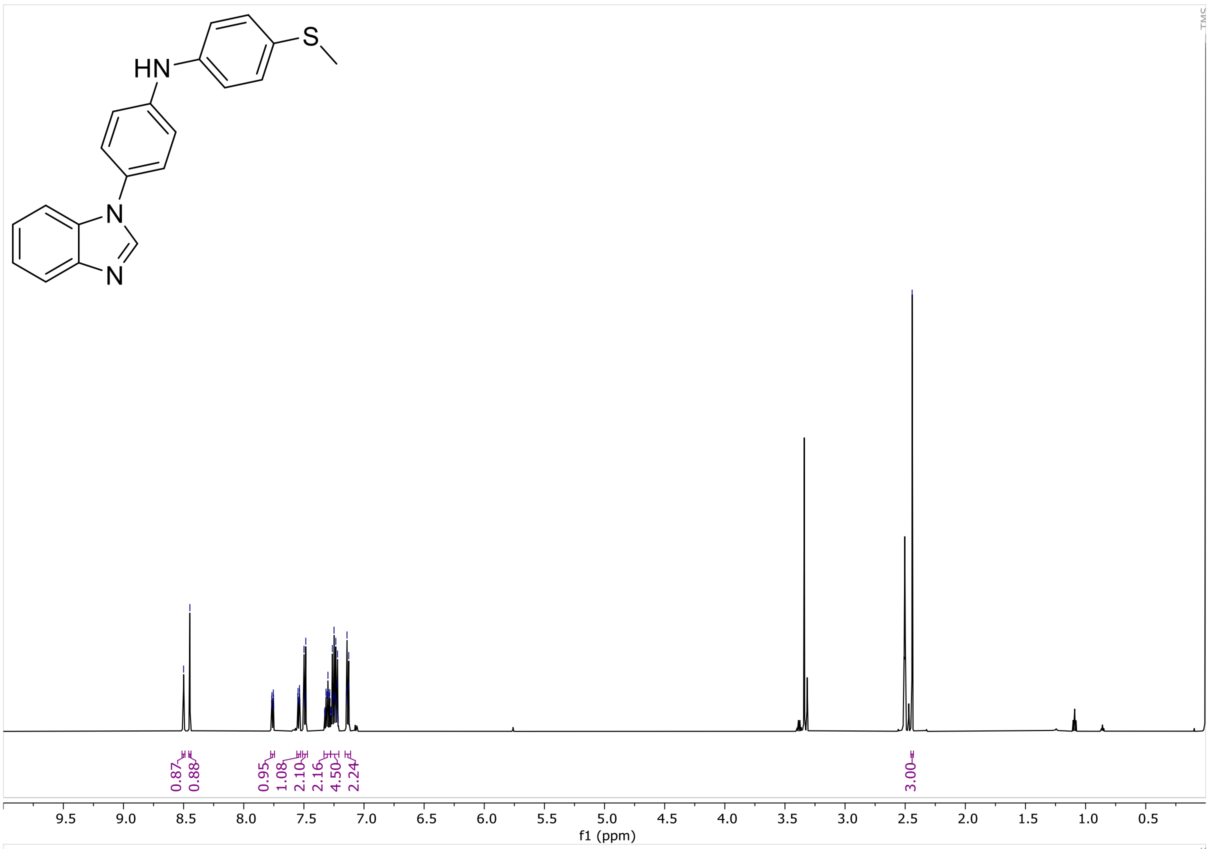

HN
S
N
N
TMS
0.87
0.88
0.95
1.08
2.10
2.16
4.50
2.24
3.00
9.5 9.0 8.5 8.0 7.5 7.0 6.5 6.0 5.5 5.0 4.5 4.0 3.5 3.0 2.5 2.0 1.5 1.0 0.5
f1 (ppm)

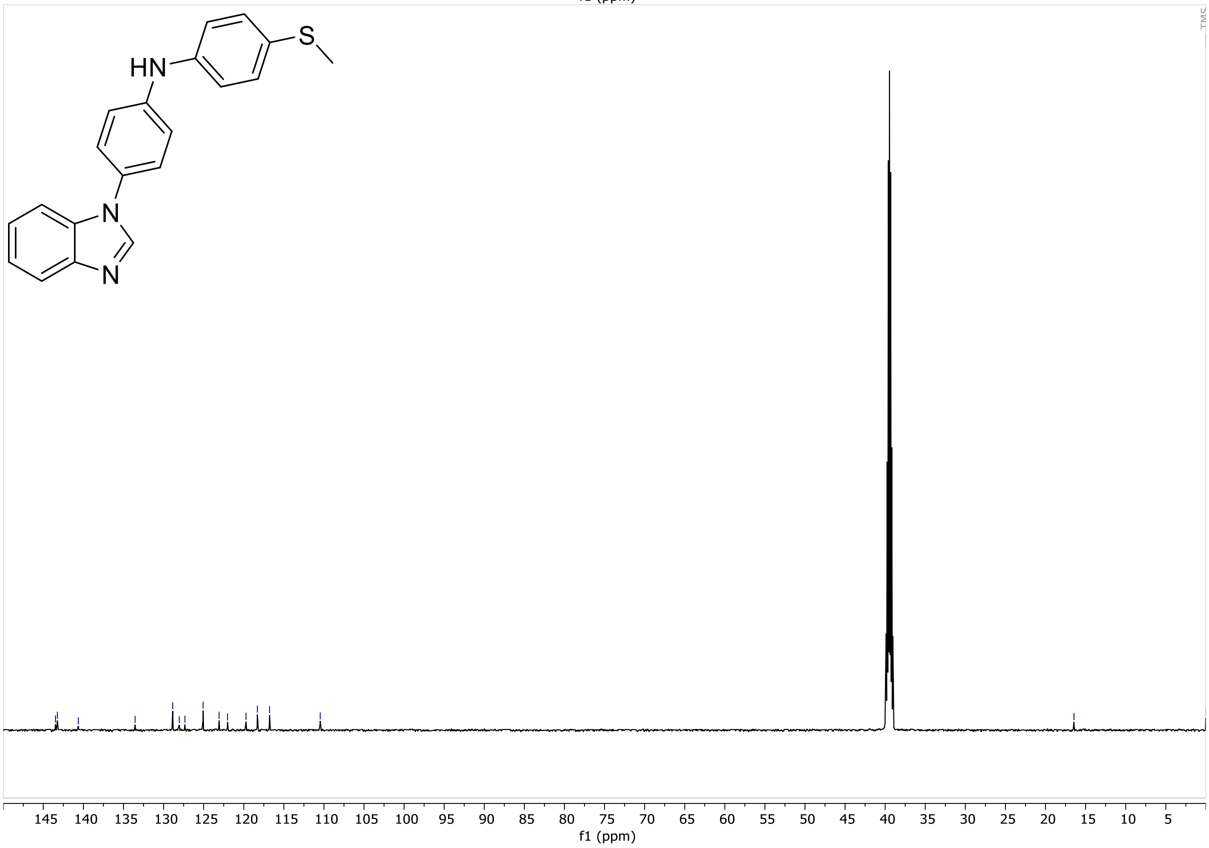

HN
S
N
N
TMS
145 140 135 130 125 120 115 110 105 100 95 90 85 80 75 70 65 60 55 50 45 40 35 30 25 20 15 10 5
f1 (ppm)

3c

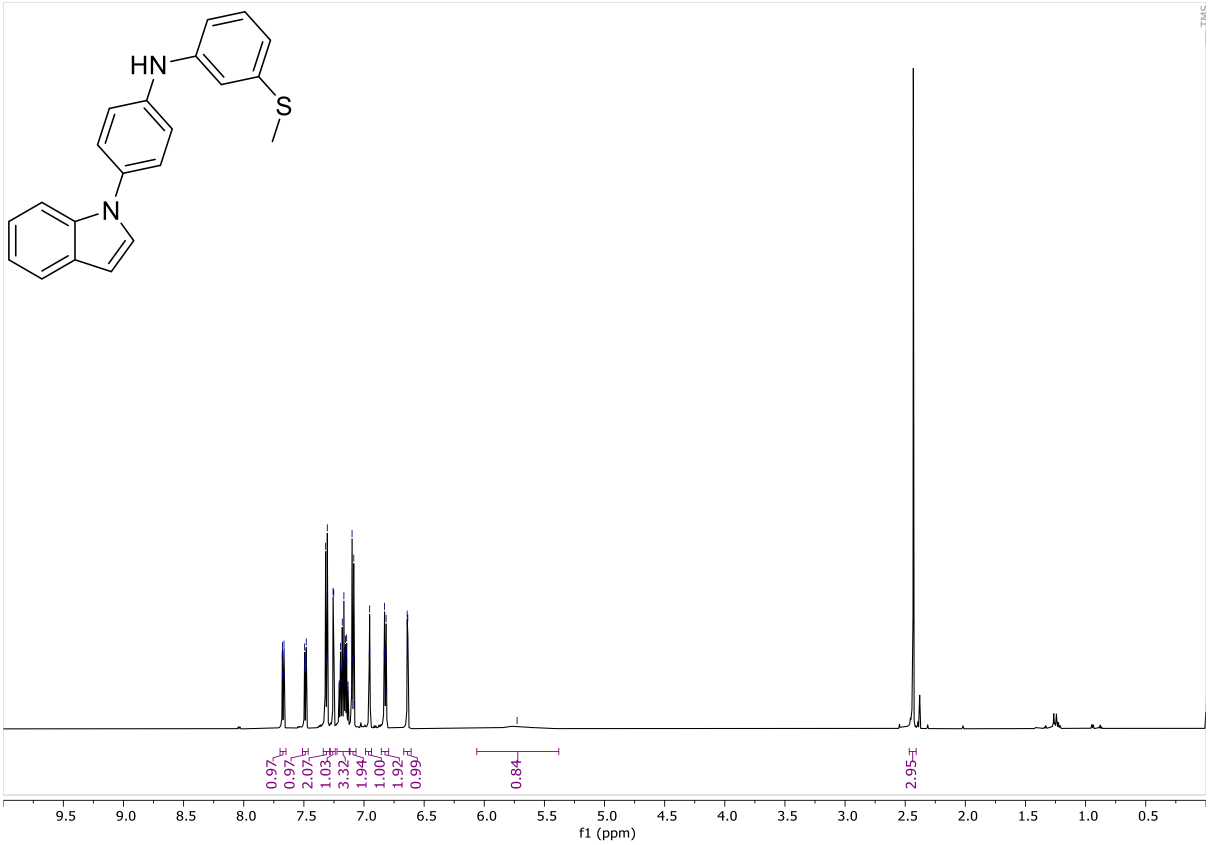

HN
S
N
0.97
0.97
2.07
1.03
3.32
1.94
1.00
1.92
0.99
0.84
2.95
9.5
9.0
8.5
8.0
7.5
7.0
6.5
6.0
5.5
5.0
4.5
4.0
3.5
3.0
2.5
2.0
1.5
1.0
0.5
f1 (ppm)

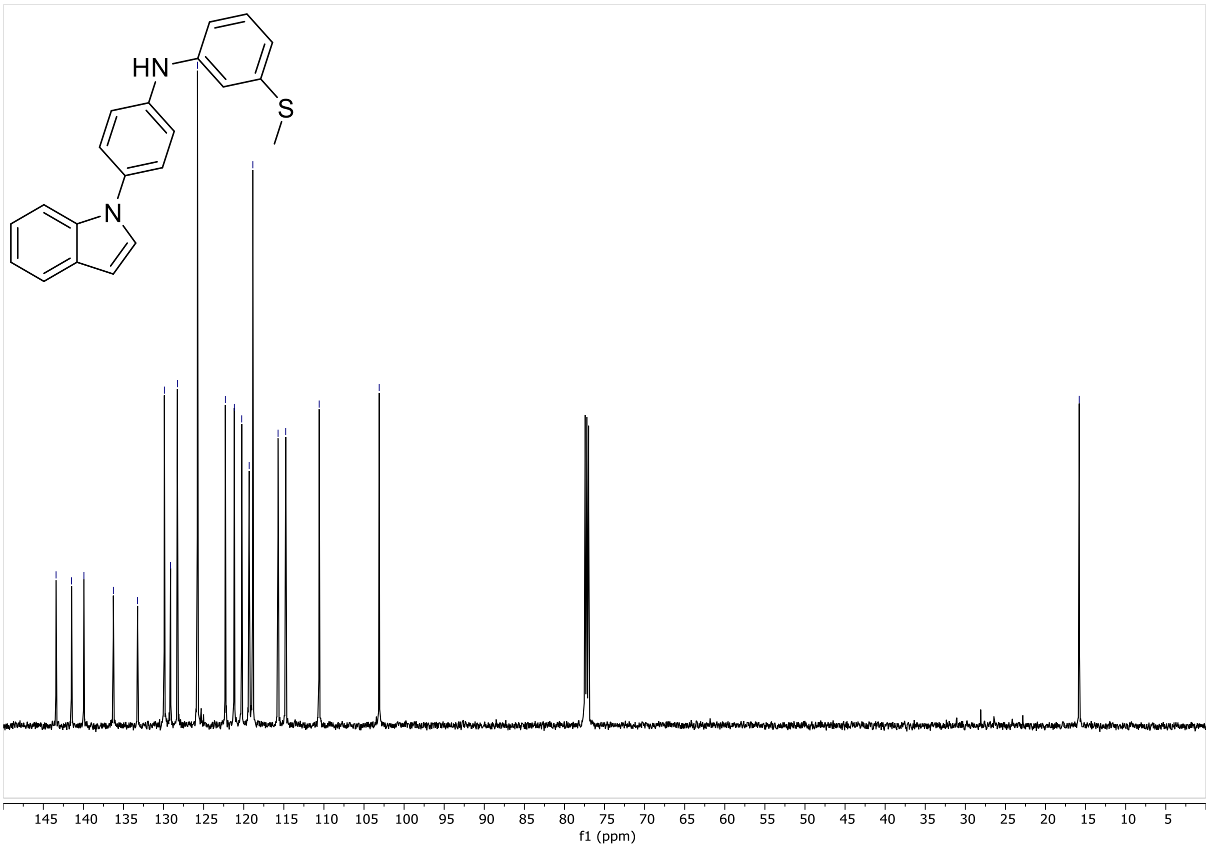

HN
S
N
145
140
135
130
125
120
115
110
105
100
95
90
85
80
75
70
65
60
55
50
45
40
35
30
25
20
15
10
5
f1 (ppm)

3d

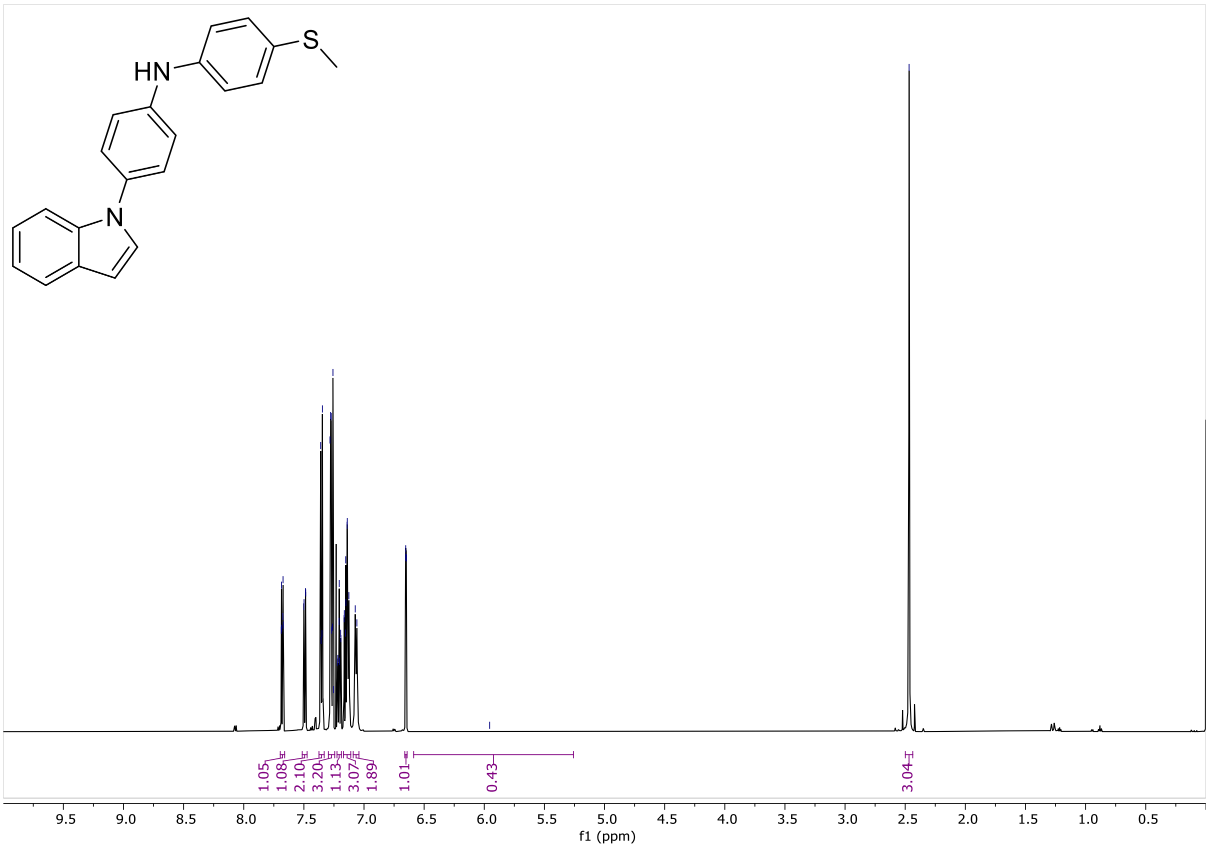
HN
S
N
1.05
1.08
2.10
3.20
1.13
3.07
1.89
1.01
0.43
3.04
9.5 9.0 8.5 8.0 7.5 7.0 6.5 6.0 5.5 5.0 4.5 4.0 3.5 3.0 2.5 2.0 1.5 1.0 0.5
f1 (ppm)

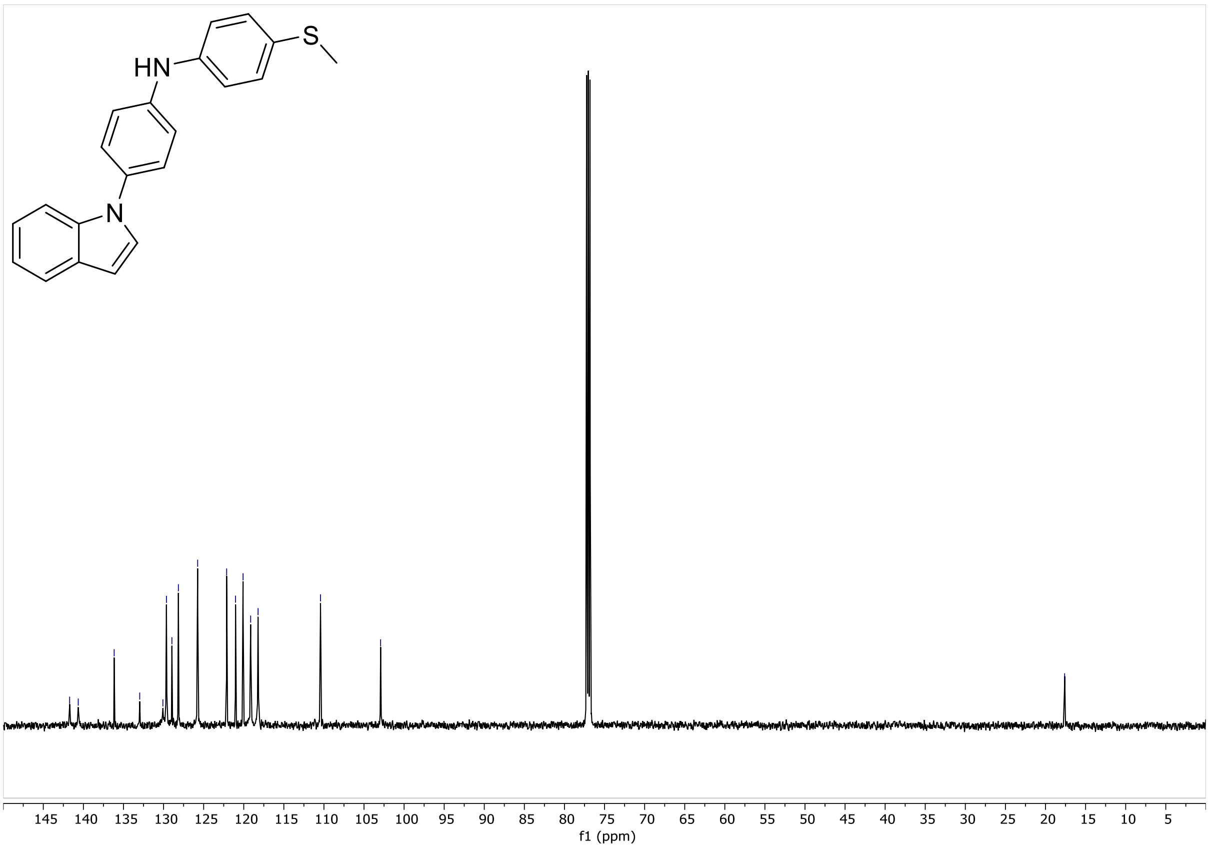
HN
S
N
145 140 135 130 125 120 115 110 105 100 95 90 85 80 75 70 65 60 55 50 45 40 35 30 25 20 15 10 5
f1 (ppm)

4a

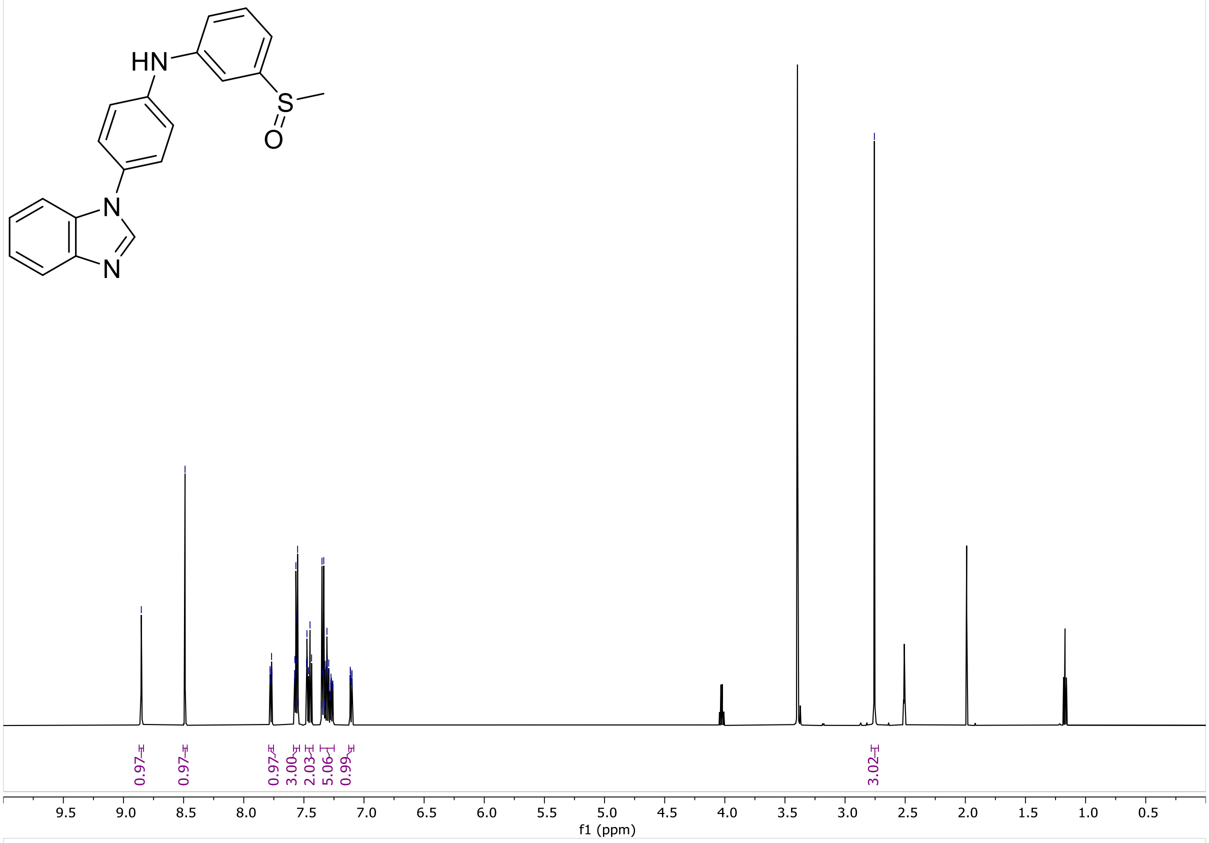
HN
S
O
N
N
0.97
0.97
0.97
3.00
2.03
5.06
0.99
3.02
9.5 9.0 8.5 8.0 7.5 7.0 6.5 6.0 5.5 5.0 4.5 4.0 3.5 3.0 2.5 2.0 1.5 1.0 0.5
f1 (ppm)

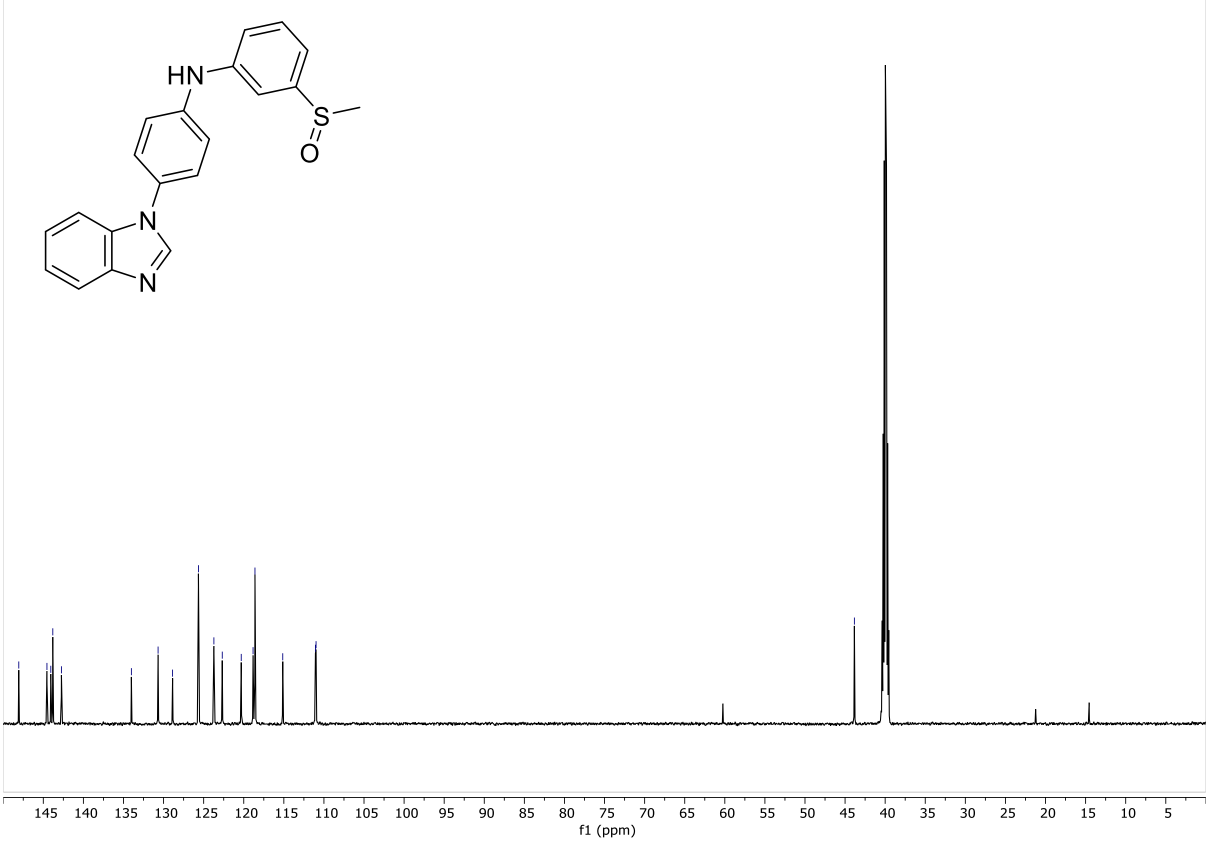
HN
S
O
N
N
145 140 135 130 125 120 115 110 105 100 95 90 85 80 75 70 65 60 55 50 45 40 35 30 25 20 15 10 5
f1 (ppm)

4b

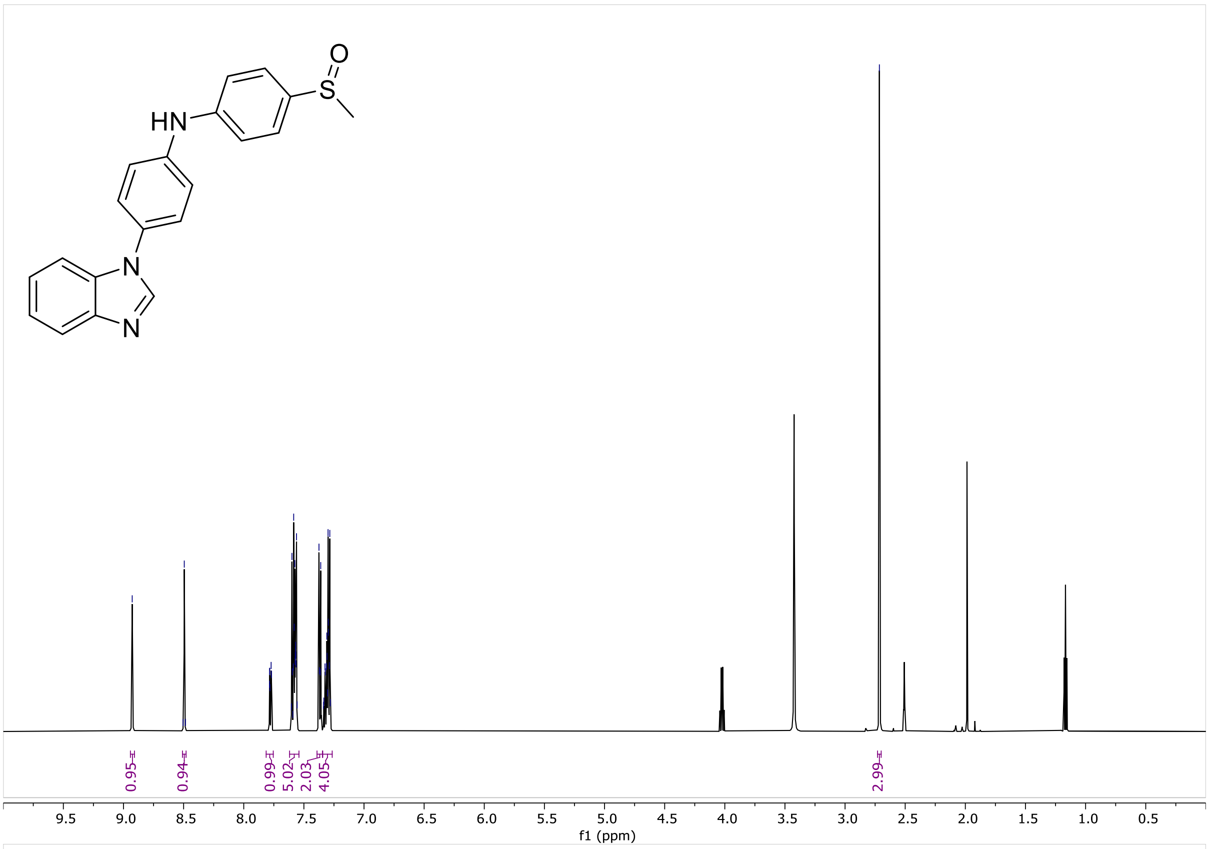

HN
O
S
N
N
0.95
0.94
0.99
5.02
2.03
4.05
2.99
9.5 9.0 8.5 8.0 7.5 7.0 6.5 6.0 5.5 5.0 4.5 4.0 3.5 3.0 2.5 2.0 1.5 1.0 0.5
f1 (ppm)

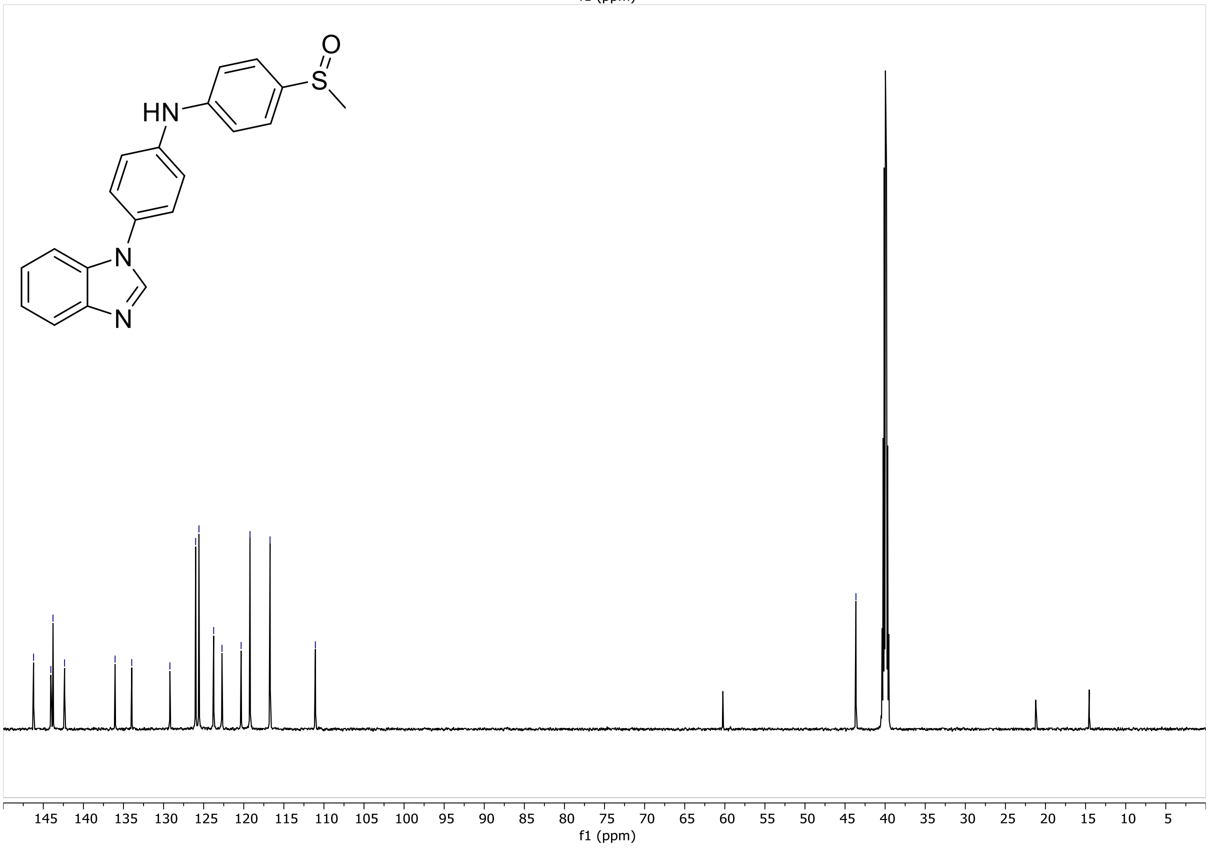

HN
O
S
N
N
145 140 135 130 125 120 115 110 105 100 95 90 85 80 75 70 65 60 55 50 45 40 35 30 25 20 15 10 5
f1 (ppm)

4c

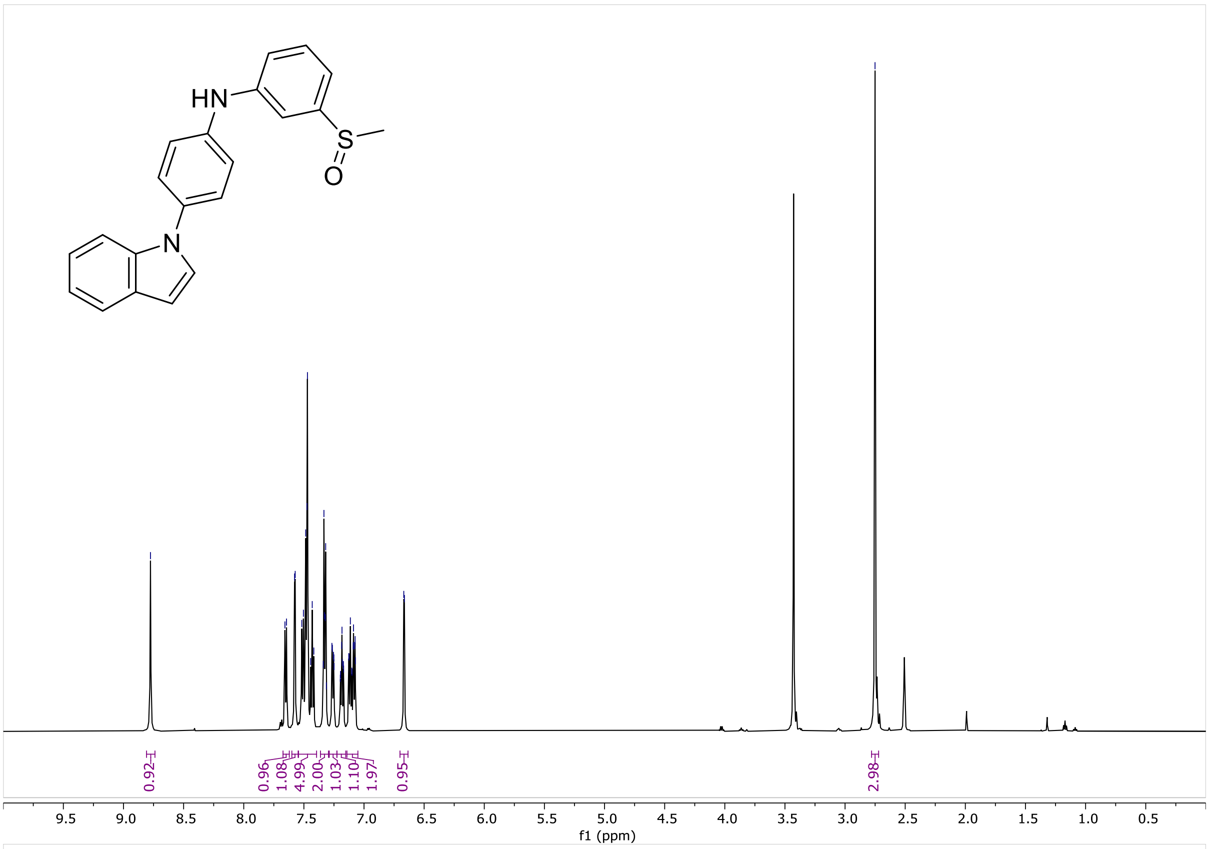

HN
N
S
O
0.92
0.96
1.08
4.99
2.00
1.03
1.10
1.97
0.95
2.98
9.5 9.0 8.5 8.0 7.5 7.0 6.5 6.0 5.5 5.0 4.5 4.0 3.5 3.0 2.5 2.0 1.5 1.0 0.5
f1 (ppm)

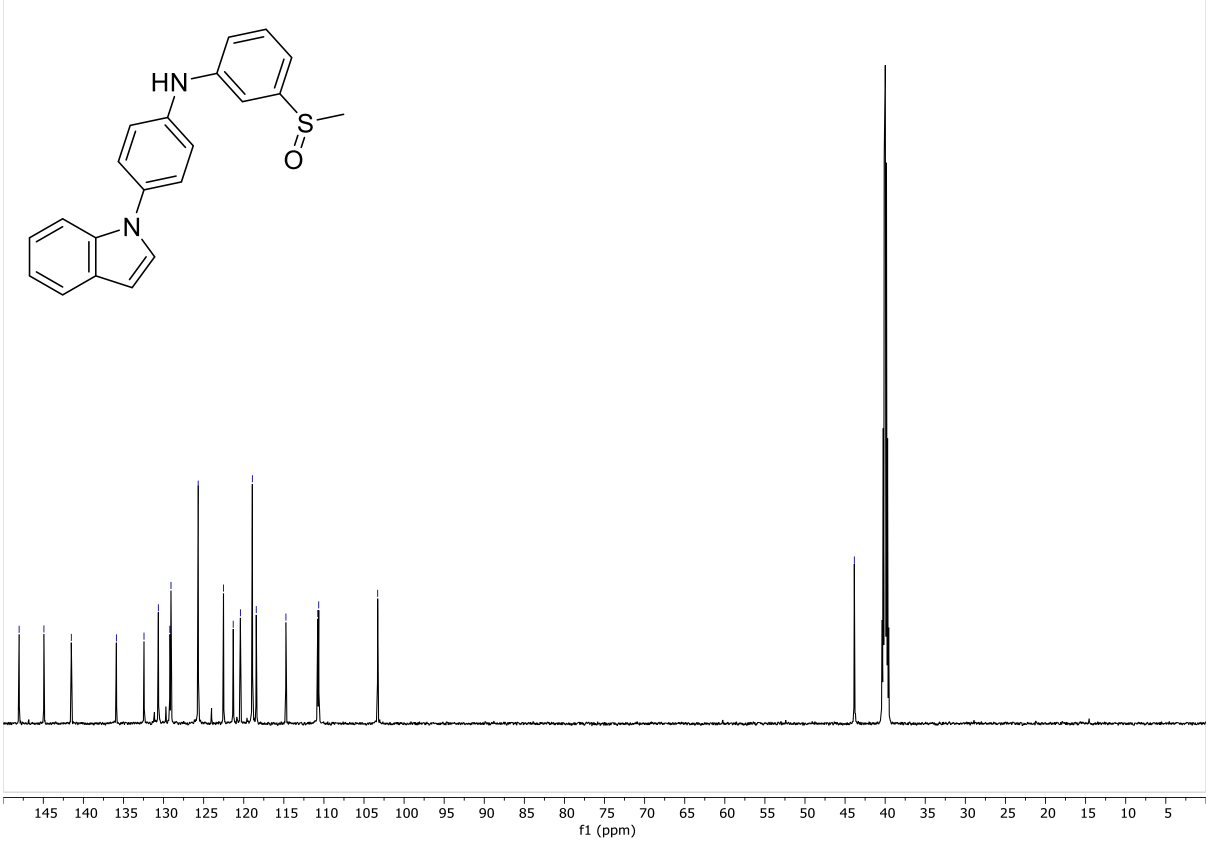

HN
N
S
O
145 140 135 130 125 120 115 110 105 100 95 90 85 80 75 70 65 60 55 50 45 40 35 30 25 20 15 10 5
f1 (ppm)

4d

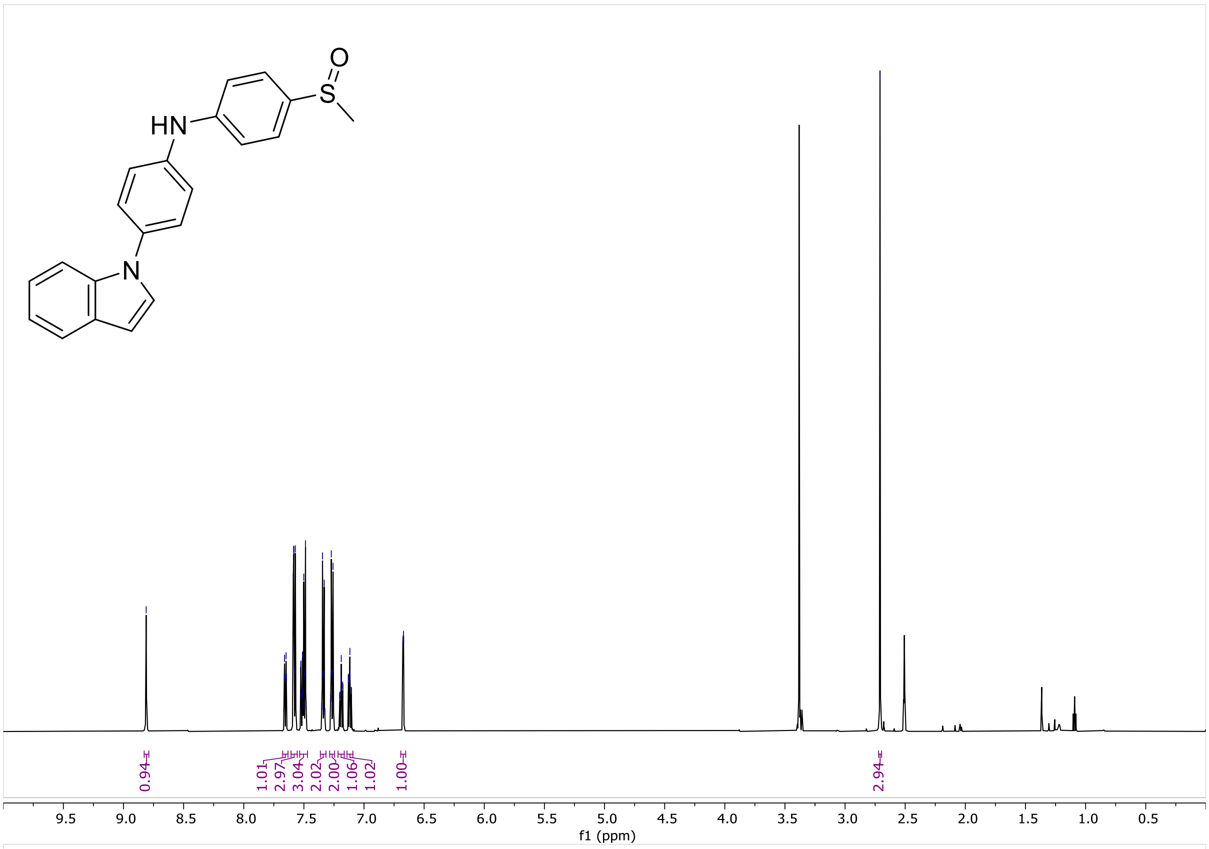
O
S
HN
N
0.94
1.01
2.97
3.04
2.02
2.00
1.06
1.02
1.00
2.94
9.5 9.0 8.5 8.0 7.5 7.0 6.5 6.0 5.5 5.0 4.5 4.0 3.5 3.0 2.5 2.0 1.5 1.0 0.5
f1 (ppm)

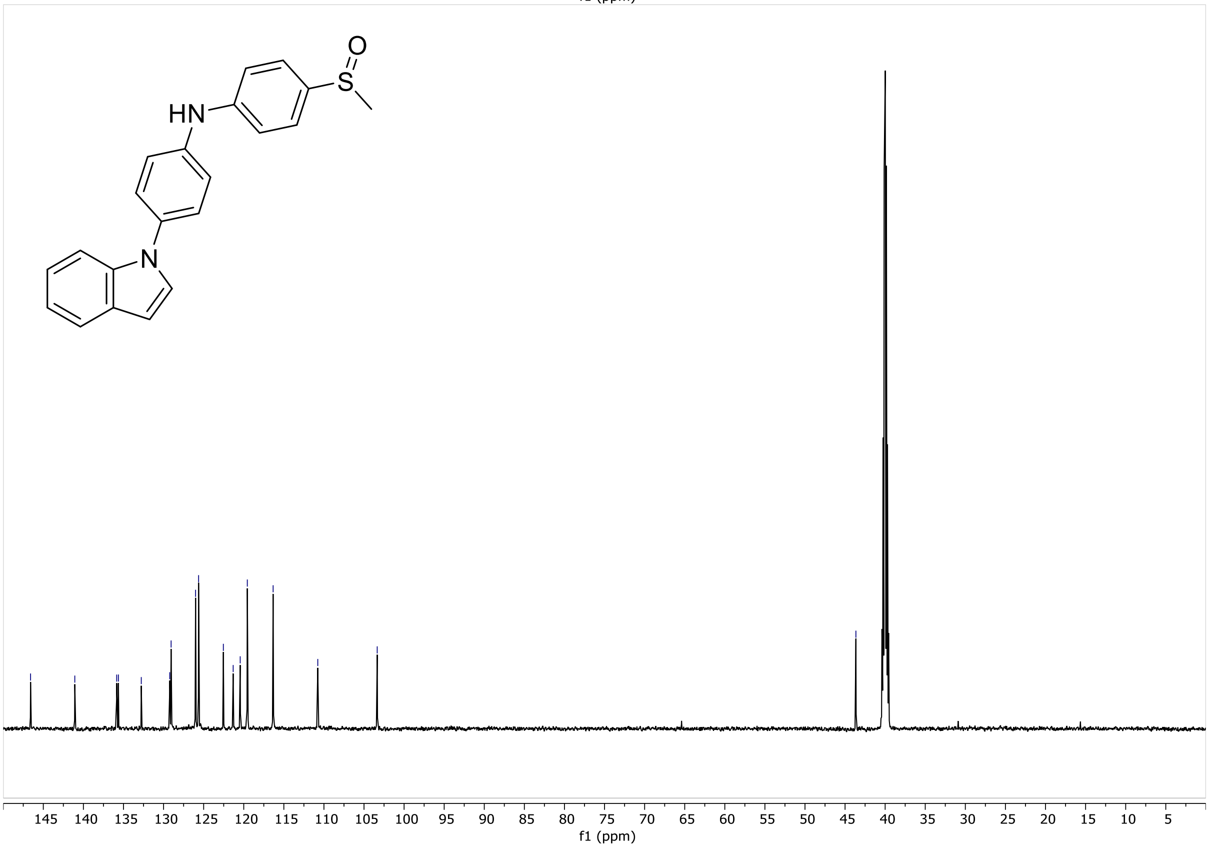
O
S
HN
N
145 140 135 130 125 120 115 110 105 100 95 90 85 80 75 70 65 60 55 50 45 40 35 30 25 20 15 10 5
f1 (ppm)

MS data

3a

Acquisition Parameter

Source Type	ESI	Ion Polarity	Positive	Set Nebulizer	3.0 Bar
Focus	Not active	Set Capillary	4500 V	Set Dry Heater	250 °C
Scan Begin	50 m/z	Set End Plate Offset	-500 V	Set Dry Gas	10.0 l/min
Scan End	1600 m/z	Set Collision Cell RF	100.0 Vpp	Set Divert Valve	Waste

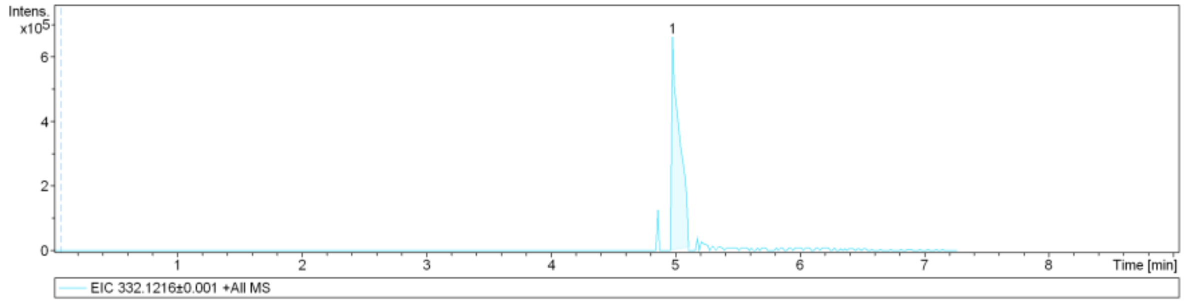

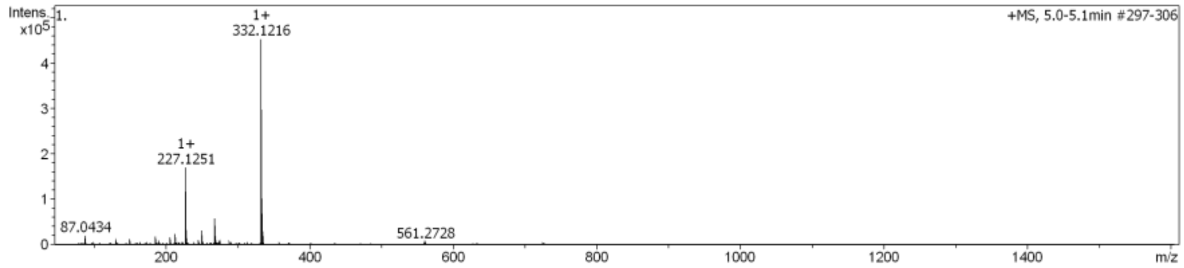

3b

Acquisition Parameter

Source Type	ESI	Ion Polarity	Positive	Set Nebulizer	3.0 Bar
Focus	Not active	Set Capillary	4500 V	Set Dry Heater	250 °C
Scan Begin	50 m/z	Set End Plate Offset	-500 V	Set Dry Gas	10.0 l/min
Scan End	1600 m/z	Set Collision Cell RF	100.0 Vpp	Set Divert Valve	Waste

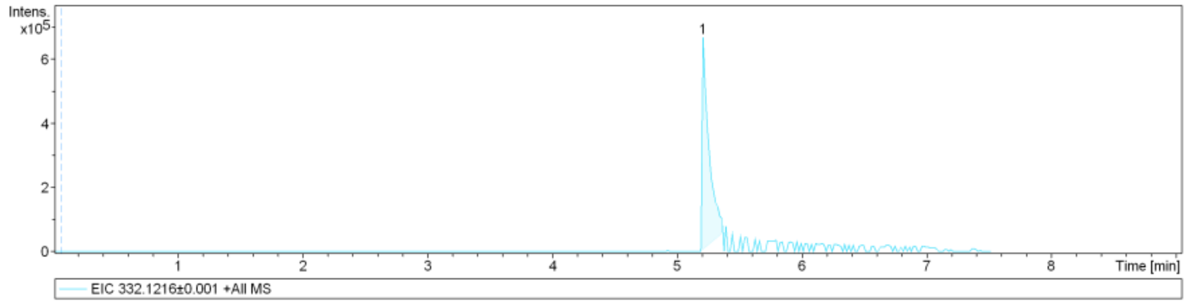

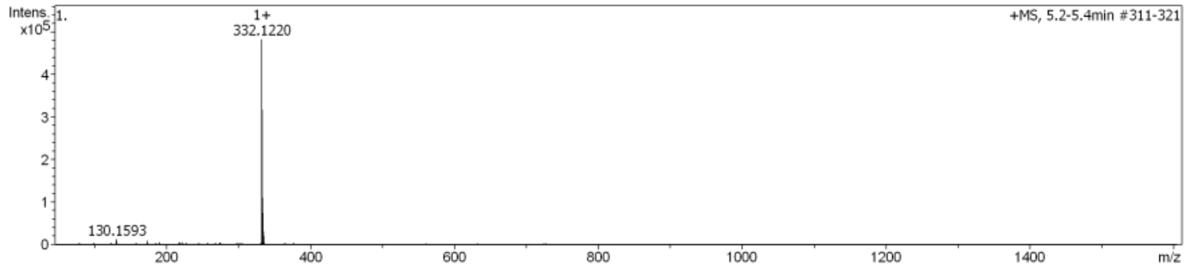

3c

Acquisition Parameter

Source Type	ESI	Ion Polarity	Positive	Set Nebulizer	3.0 Bar
Focus	Not active	Set Capillary	4500 V	Set Dry Heater	250 °C
Scan Begin	50 m/z	Set End Plate Offset	-500 V	Set Dry Gas	10.0 l/min
Scan End	1600 m/z	Set Collision Cell RF	100.0 Vpp	Set Divert Valve	Waste

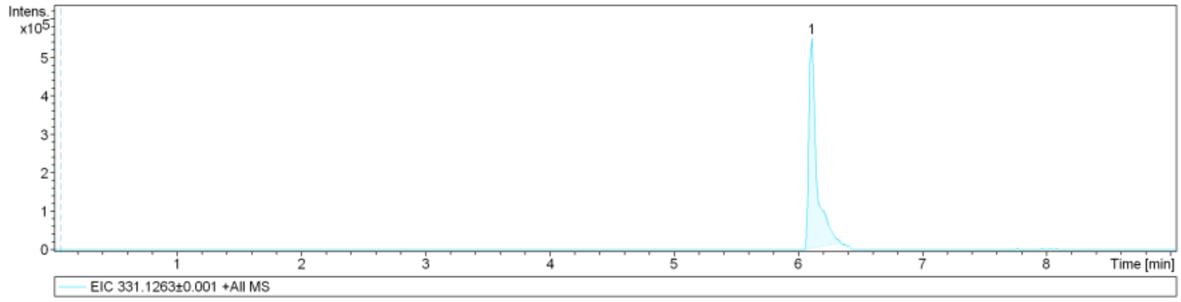

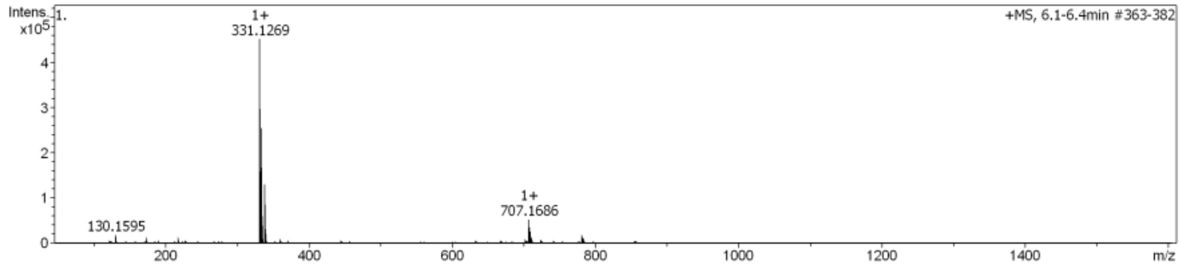

3d

Acquisition Parameter

Source Type	ESI	Ion Polarity	Positive	Set Nebulizer	3.0 Bar
Focus	Not active	Set Capillary	4500 V	Set Dry Heater	250 °C
Scan Begin	50 m/z	Set End Plate Offset	-500 V	Set Dry Gas	10.0 l/min
Scan End	1600 m/z	Set Collision Cell RF	100.0 Vpp	Set Divert Valve	Waste

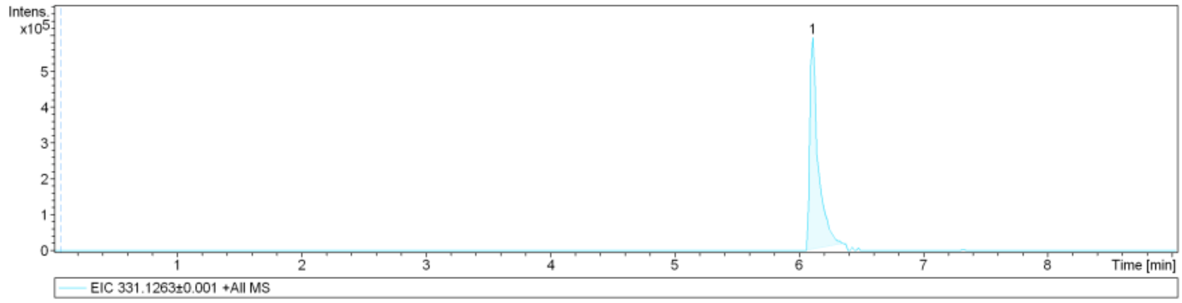

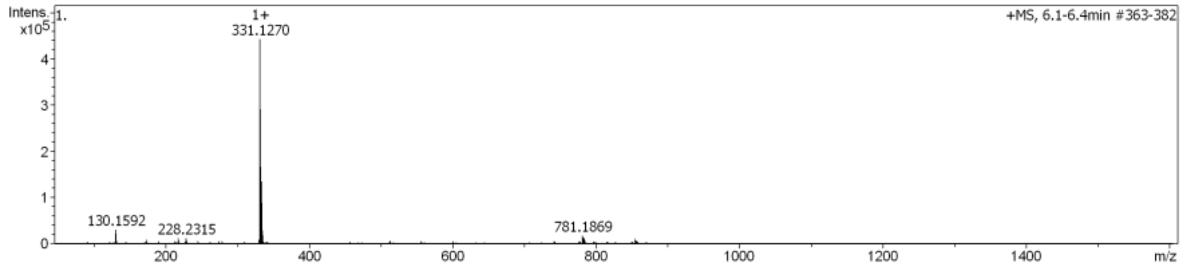

4a

Acquisition Parameter

Source Type	ESI	Ion Polarity	Positive	Set Nebulizer	3.0 Bar
Focus	Not active	Set Capillary	4500 V	Set Dry Heater	250 °C
Scan Begin	50 m/z	Set End Plate Offset	-500 V	Set Dry Gas	10.0 l/min
Scan End	1600 m/z	Set Collision Cell RF	100.0 Vpp	Set Divert Valve	Waste

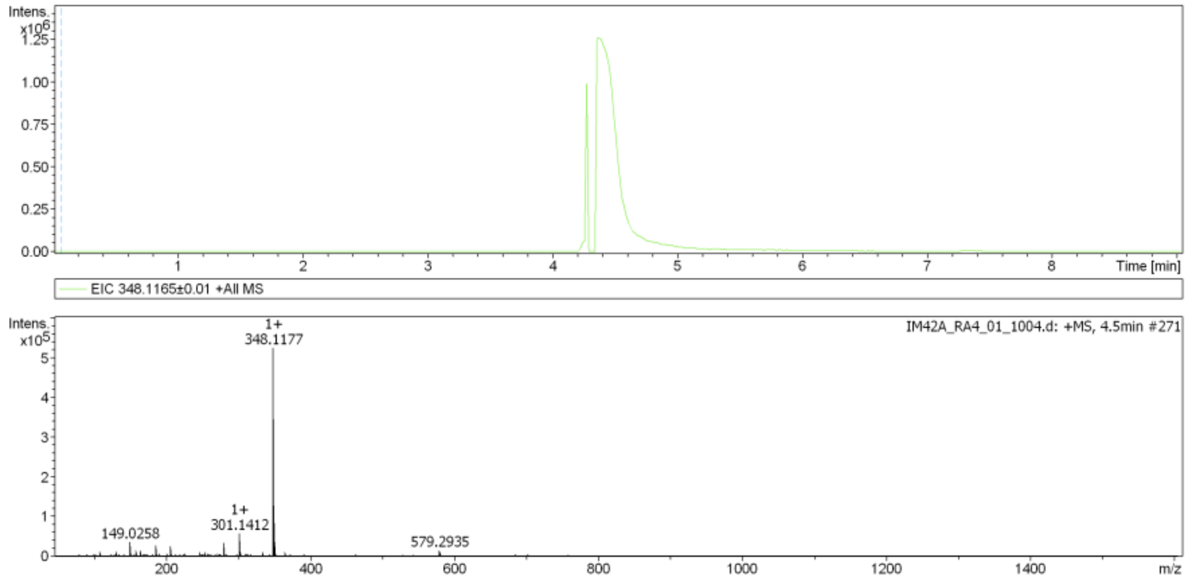

4b

Acquisition Parameter

Source Type	ESI	Ion Polarity	Positive	Set Nebulizer	3.0 Bar
Focus	Not active	Set Capillary	4500 V	Set Dry Heater	250 °C
Scan Begin	50 m/z	Set End Plate Offset	-500 V	Set Dry Gas	10.0 l/min
Scan End	1600 m/z	Set Collision Cell RF	100.0 Vpp	Set Divert Valve	Waste

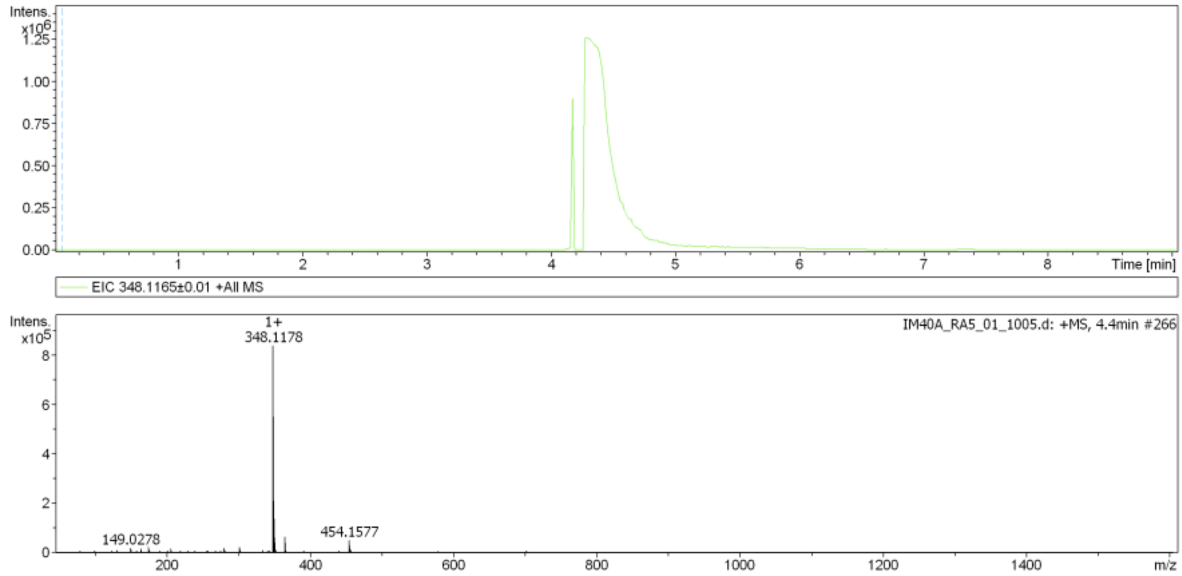

4c

Acquisition Parameter

Source Type	ESI	Ion Polarity	Positive	Set Nebulizer	3.0 Bar
Focus	Not active	Set Capillary	4500 V	Set Dry Heater	250 °C
Scan Begin	50 m/z	Set End Plate Offset	-500 V	Set Dry Gas	10.0 l/min
Scan End	1600 m/z	Set Collision Cell RF	100.0 Vpp	Set Divert Valve	Waste

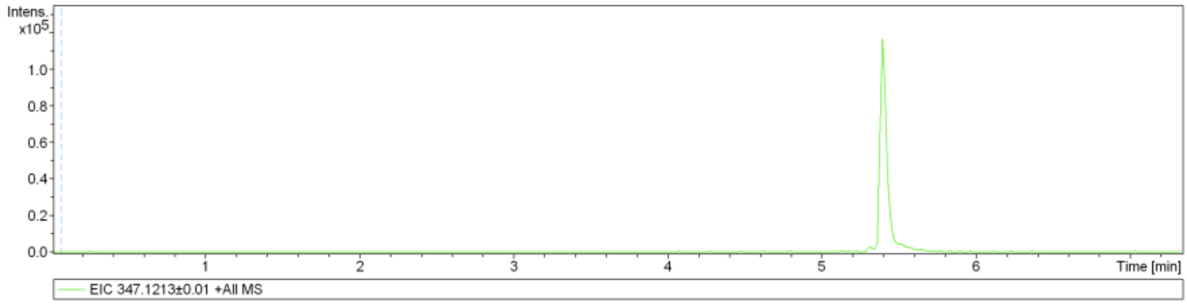

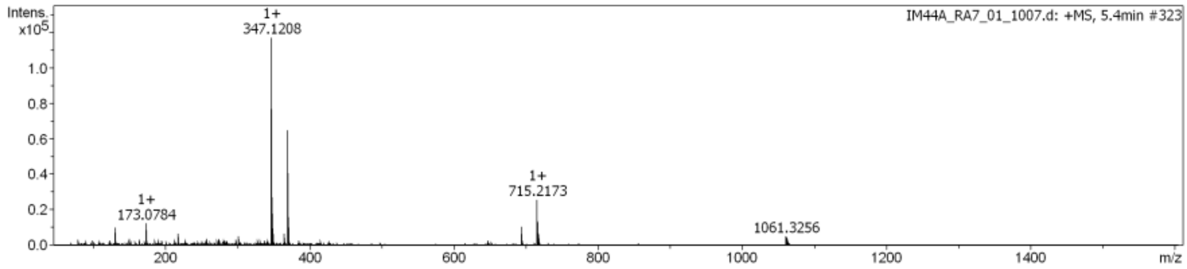

4d

Acquisition Parameter

Source Type	ESI	Ion Polarity	Positive	Set Nebulizer	3.0 Bar
Focus	Not active	Set Capillary	4500 V	Set Dry Heater	250 °C
Scan Begin	50 m/z	Set End Plate Offset	-500 V	Set Dry Gas	10.0 l/min
Scan End	1600 m/z	Set Collision Cell RF	100.0 Vpp	Set Divert Valve	Waste

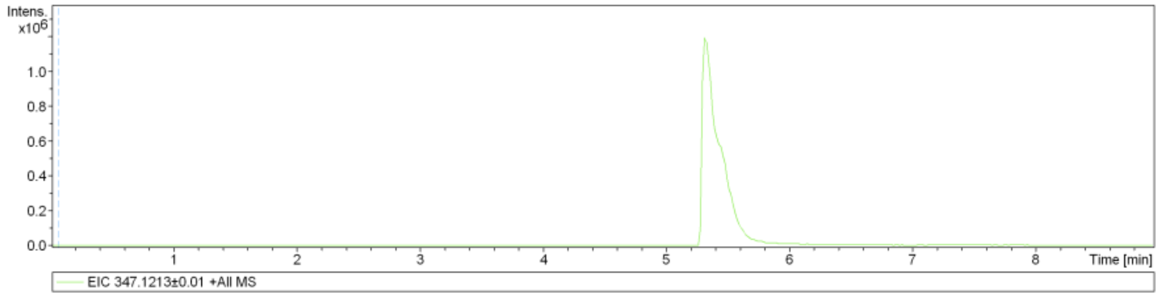

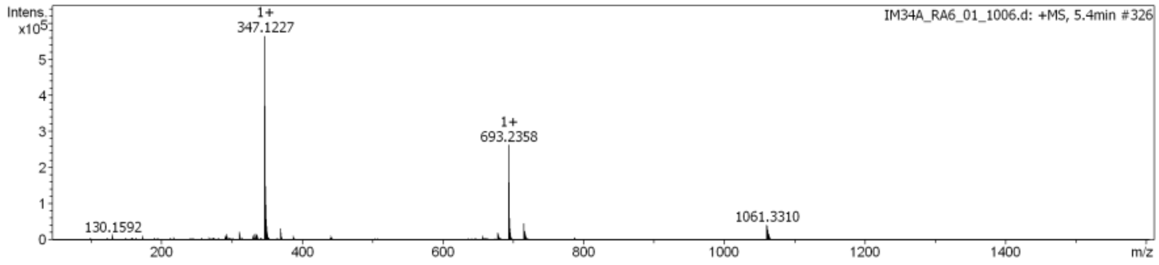

HPLC data

3a

Chromatogram

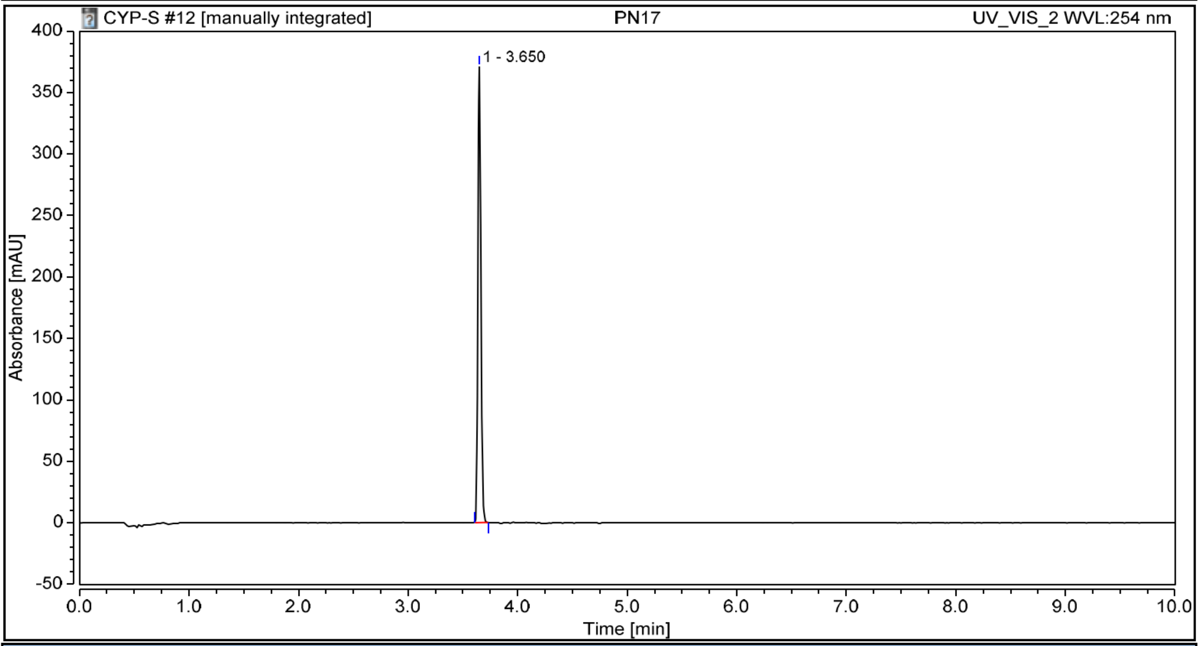

Integration Results

No.	Peak Name	Retention Time min	Area mAU*min	Height mAU	Relative Area %	Relative Height %	Amount
n.a. 1	Component 1	n.a. 3.65	n.a. 11.640	n.a. 370.777	n.a. 100.00	n.a. 100.00	n.a. n.a.
**Total:**			**11.64**	**370.78**	**100.0**	**100.0**	

3b

Chromatogram

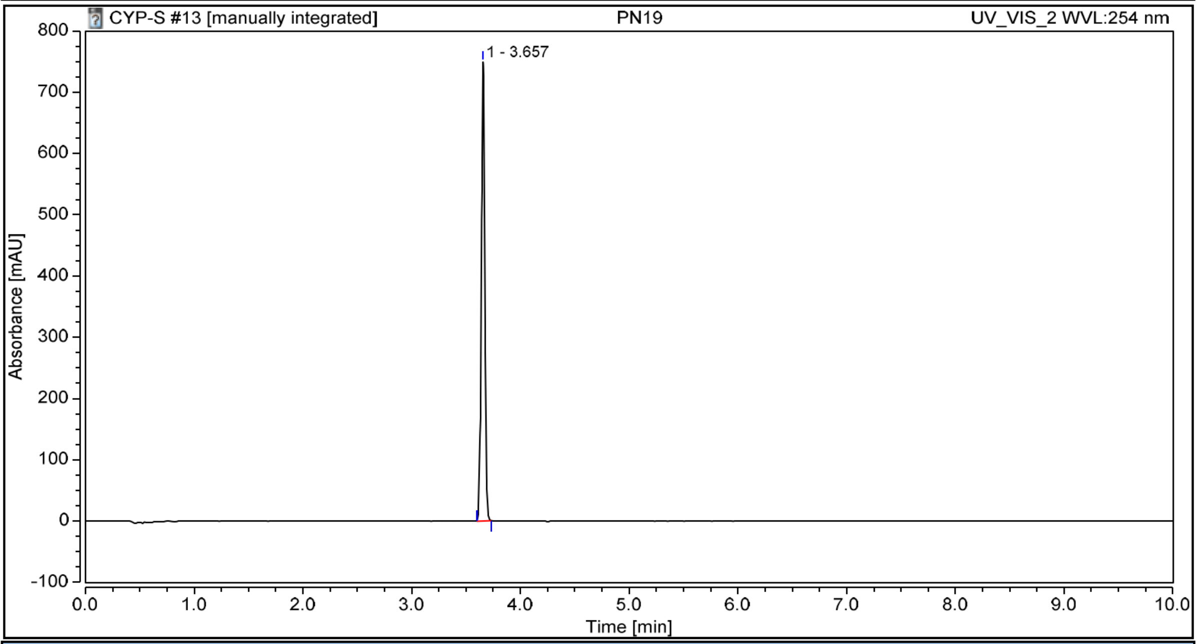

Integration Results

No.	Peak Name	Retention Time min	Area mAU*min	Height mAU	Relative Area %	Relative Height %	Amount
n.a. 1	Component 1	n.a. 3.66	n.a. 27.677	n.a. 749.084	n.a. 100.00	n.a. 100.00	n.a. n.a.
**Total:**			**27.68**	**749.08**	**100.0**	**100.0**	

3c

Chromatogram

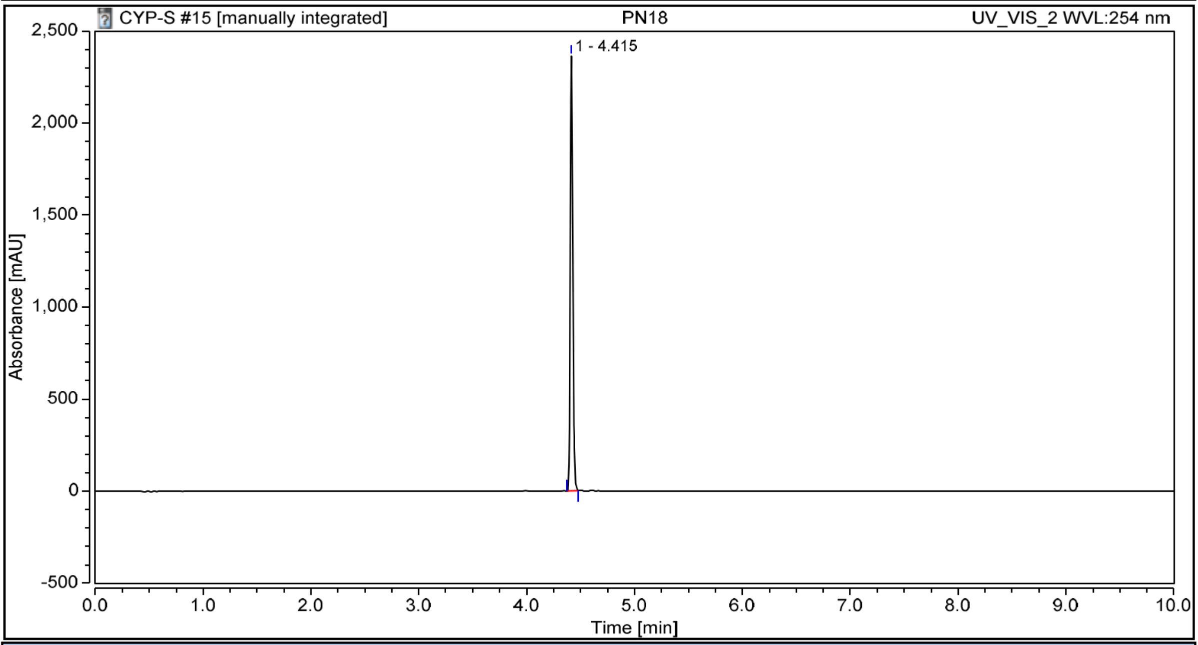

Integration Results

No.	Peak Name	Retention Time<br>min	Area<br>mAU*min	Height<br>mAU	Relative Area<br>%	Relative Height<br>%	Amount
n.a.	Component 1	n.a.	n.a.	n.a.	n.a.	n.a.	n.a.
1		4.42	62.874	2363.031	100.00	100.00	n.a.
**Total:**			**62.87**	**2363.03**	**100.0**	**100.0**	

3d

Chromatogram

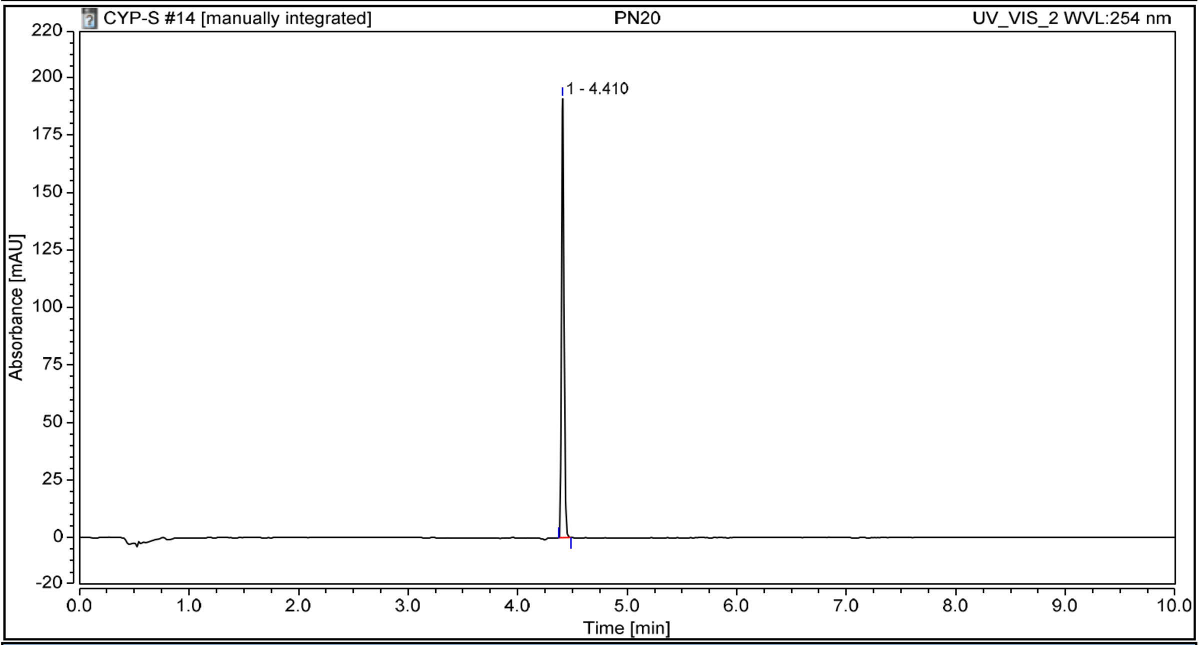

Integration Results

No.	Peak Name	Retention Time<br>min	Area<br>mAU*min	Height<br>mAU	Relative Area<br>%	Relative Height<br>%	Amount
n.a.	Component 1	n.a.	n.a.	n.a.	n.a.	n.a.	n.a.
1		4.41	5.036	190.817	100.00	100.00	n.a.
**Total:**			**5.04**	**190.82**	**100.0**	**100.0**	

4a

Chromatogram

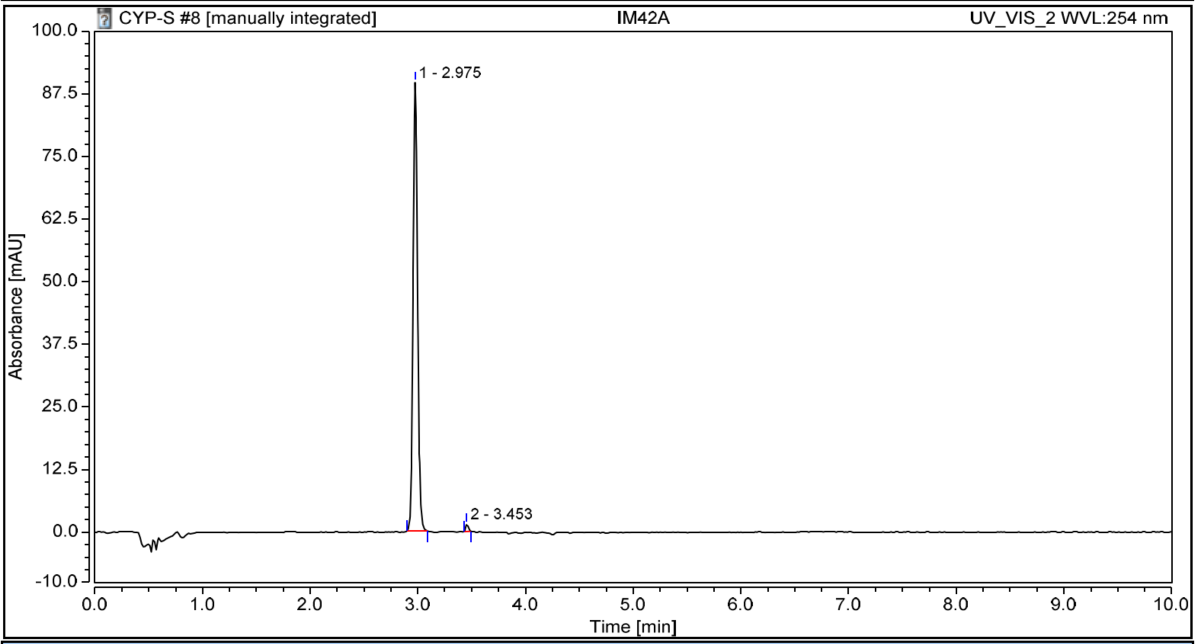

Integration Results

No.	Peak Name	Retention Time<br>min	Area<br>mAU*min	Height<br>mAU	Relative Area<br>%	Relative Height<br>%	Amount
n.a.	Component 1	n.a.	n.a.	n.a.	n.a.	n.a.	n.a.
1		2.98	4.373	89.523	99.01	98.39	n.a.
2		3.45	0.044	1.463	0.99	1.61	n.a.
**Total:**			**4.42**	**90.99**	**100.0**	**100.0**	

4b

Chromatogram

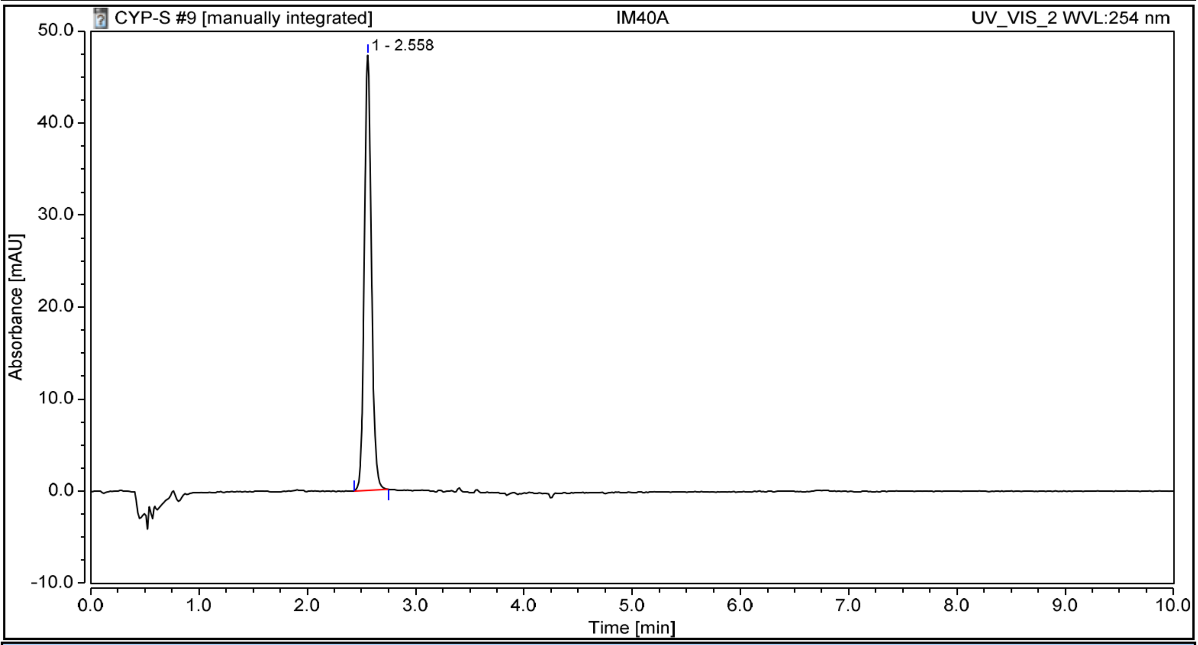

Integration Results

No.	Peak Name	Retention Time<br>min	Area<br>mAU*min	Height<br>mAU	Relative Area<br>%	Relative Height<br>%	Amount
n.a.	Component 1	n.a.	n.a.	n.a.	n.a.	n.a.	n.a.
1		2.56	3.550	47.281	100.00	100.00	n.a.
**Total:**			**3.55**	**47.28**	**100.0**	**100.0**	

4c

Chromatogram

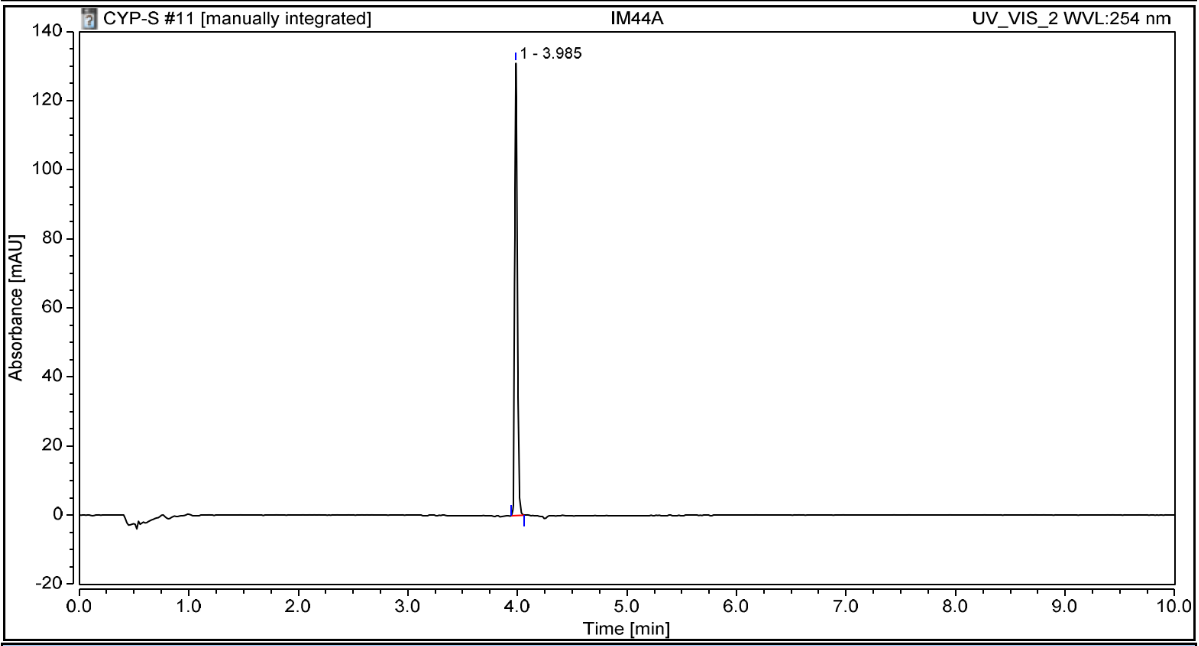

Integration Results

No.	Peak Name	Retention Time min	Area mAU*min	Height mAU	Relative Area %	Relative Height %	Amount
n.a. 1	Component 1	n.a. 3.99	n.a. 3.801	n.a. 130.972	n.a. 100.00	n.a. 100.00	n.a. n.a.
**Total:**			**3.80**	**130.97**	**100.0**	**100.0**	

4d

Chromatogram

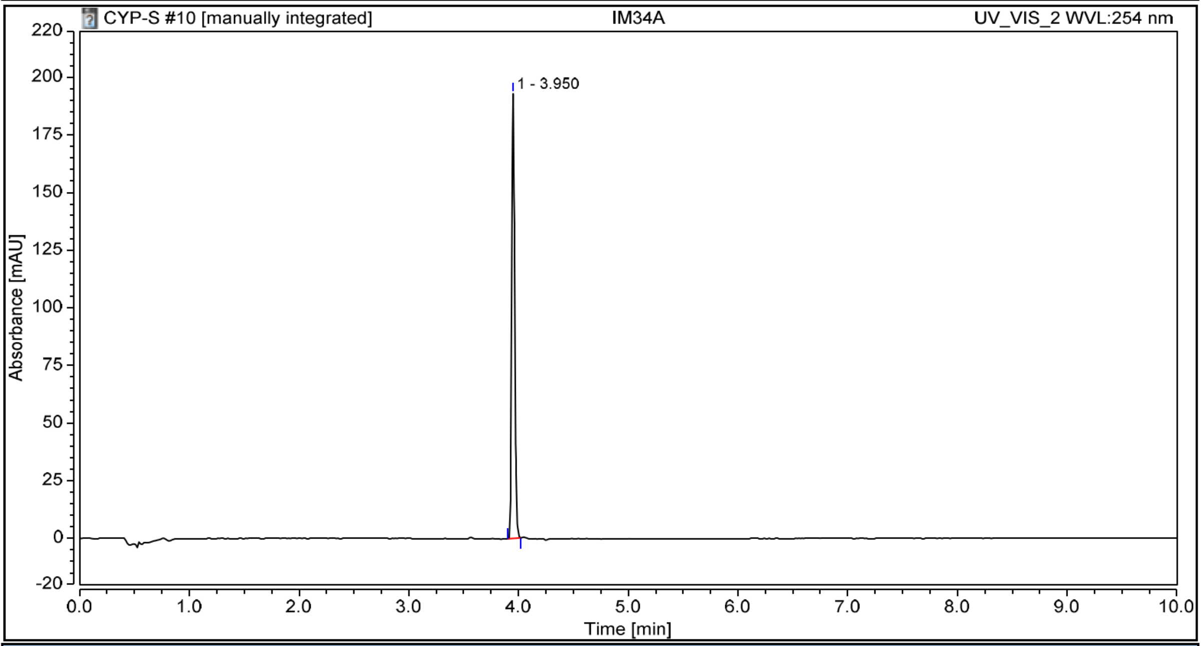

Integration Results

No.	Peak Name	Retention Time min	Area mAU*min	Height mAU	Relative Area %	Relative Height %	Amount
n.a. 1	Component 1	n.a. 3.95	n.a. 5.664	n.a. 192.877	n.a. 100.00	n.a. 100.00	n.a. n.a.
**Total:**			**5.66**	**192.88**	**100.0**	**100.0**	
